# Filling Roots of Teeth

**Published:** 1880-11

**Authors:** Samuel D. Rambo

**Affiliations:** Rio De Janeiro, Brazil


ARTICLE V.

### FILLING ROOTS OF TEETH.

BY SAMUEL D. RAMBO, D. D. S., RIO DE JANEIRO, BRAZIL

The subject of filling roots of teeth has often been discussed in Dental Associations, has been written and re-written upon, each advocating their favorite mode of accomplishing this operation. I have employed all of their systems of fang filling. My experience with gold has not been entirely satisfactory, for this reason; there is danger of carrying the gold beyond the apex, although length of canal has been taken. The foramen of some roots are very large at the apex, and that being the case, it is not a very difficult matter to force the gold beyond its stopping place. A line beyond is sufficient to give trouble. I have extracted teeth where the gold had been forced through the apex, causing constant irritation, and therefore always an abscess. Gutta percha is objectionable for same reason. The oxy chlorides in my opinion, are the most inferior of them all; being an escharotic, they destroy the periosteum around the apex of the root. It disintegrates, becomes soft and absorbs the secretions, and we then have almost the same thing as decomposed nerve in the canal. The Phosphoric filling is better, since it is not a cautery, and remains hard, but by employing it, we run a risk of forcing the material through upon the soft parts. I have driven wood and whale bone into the canals of fangs, but was not satisfied with the adaptation. I have abandoned all of these, and have taken to filling the apex with lead needles. I take a piece of sheet lead and cut a strip from it, and pass through a draw plate until it is reduced to the size of a medium pin, cut the wire into pieces one inch long and roll one end to fit foramen at apex. If I find in passing it up, it goes through, which I ascertain from the pain produced, I take it out and cut the point off a little, and try it again; when I find it has closed the apex exactly, which I know from the touch, I pass down by the side of it, a nerve plugger, same shape as lead needle introduced, making room for another wire, and so on until I find the first third of root filled. I then fill the middle part with gutta percha, cutting the wires off as far up as I can, otherwise the lead might discolor the tooth at the margin of the gums; I then fill the last third of root with the white phosphoric filling, to prevent the tooth from turning dark. Before introducing the lead wires I moisten them with phenic acid or creosote. Lead is less irritating in the flesh than any of the metals. I have found buck shot in deer that had long since healed over, and no sign of inflammation or ulcers. For the reason that flesh will kindly heal around lead, I think it the best material that can be employed for filling the fangs of teeth.

